# Collective intelligence in fingerprint analysis

**DOI:** 10.1186/s41235-020-00223-8

**Published:** 2020-05-19

**Authors:** Jason M. Tangen, Kirsty M. Kent, Rachel A. Searston

**Affiliations:** 1 School of Psychology, The University of Queensland, St Lucia, 4072 Queensland Australia; 2 School of Psychology, The University of Adelaide, Adelaide, 5005 South Australia Australia

**Keywords:** Collective intelligence, Wisdom of crowds, Expertise, Fingerprints, Forensic science

## Abstract

When a fingerprint is located at a crime scene, a human examiner is counted upon to manually compare this print to those stored in a database. Several experiments have now shown that these professional analysts are highly accurate, but not infallible, much like other fields that involve high-stakes decision-making. One method to offset mistakes in these safety-critical domains is to distribute these important decisions to groups of raters who independently assess the same information. This redundancy in the system allows it to continue operating effectively even in the face of rare and random errors. Here, we extend this “wisdom of crowds” approach to fingerprint analysis by comparing the performance of individuals to crowds of professional analysts. We replicate the previous findings that individual experts greatly outperform individual novices, particularly in their false-positive rate, but they do make mistakes. When we pool the decisions of small groups of experts by selecting the decision of the majority, however, their false-positive rate decreases by up to 8% and their false-negative rate decreases by up to 12%. Pooling the decisions of novices results in a similar drop in false negatives, but increases their false-positive rate by up to 11%. Aggregating people’s judgements by selecting the majority decision performs better than selecting the decision of the most confident or the most experienced rater. Our results show that combining independent judgements from small groups of fingerprint analysts can improve their performance and prevent these mistakes from entering courts.

## Public Significance Statement

Several reports by peak scientific and regulatory bodies have been roundly critical of the dearth of evidence supporting traditional forensic methods and practices such as fingerprint analysis. In response to these criticisms, a number of experiments have now been conducted, demonstrating that professional fingerprint analysts are impressively accurate compared to novices when distinguishing between crime-scene prints from the same and different sources—but they still make mistakes. These mistakes are unavoidable, even in other high stakes, safety-critical domains such as medicine, aviation, or nuclear power. The aim, then, is to build safeguards into these systems that mitigate the impact of these mistakes in practice. In this experiment, we examine one such countermeasure, which exploits the collective intelligence of groups of professional fingerprint analysts. Our results show that pooling the decisions of small, independent groups of examiners can substantially boost the overall performance of these crowds and reduce the influence of errors. Integrating collective intelligence processes into existing forensic identification and verification systems could play a significant role—alongside effective training methods and evidence-based practices—in developing reliable and resilient systems to ensure the rule of law is justly applied.

## Collective intelligence in fingerprint analysis

When a fingerprint is recovered from a crime scene, a computer algorithm is used to compare the print to tens of millions of prints stored in a database. The algorithm then returns a list of potential candidates ranked from the most to the least similar. It is up to a human examiner to work through this list, comparing the overall pattern and flow of the prints as well as the fine details in each, such as ridge endings, bifurcations, contours, islands, dots, breaks, creases, pores, and enclosures. If the examiner has identified a sufficient number of corresponding features to be confident that the two prints came from the same person, then this final “same source” decision is logged into the computer system, which is then typically “verified” by a second examiner who—depending on their jurisdiction—may or may not be blind to the initial examiner’s decision (Thompson, Black, Jain, & Kadane, 2017).

Despite the fact that fingerprint examiners have been shown to perform exceptionally well (Searston & Tangen, 2017a; Searston & Tangen, 2017b; Tangen, Thompson, & McCarthy, 2011; Thompson & Tangen, 2014; Ulery, Hicklin, Buscaglia, & Roberts, 2011), errors have occurred in the past (Cole, 2005). A correct identification can mean the difference between exonerating a criminal or convicting an innocent person. It is clear that forensic analysts are working hard to capture criminals and uphold civil liberties; they have a very high workload, relentlessly coding evidence, supporting detectives, searching and maintaining databases, writing reports, and testifying as expert witnesses. It is also clear that—despite everyone’s best efforts—mistakes happen and will continue to happen, even when people’s lives are at stake. In medical diagnostics for example, roughly 200,000 patients die from preventable medical errors each year (James, 2013), and 5% of autopsies reveal lethal diagnostic errors that could have been averted (Shojania, Burton, McDonald, & Goldman, 2003). One of the main conclusions of an authoritative report on these errors by the National Institute of Medicine was that these mistakes do not result from individual recklessness or the actions of a particular group. Instead, it is important to design resilient systems that identify and enhance the positive capacities of people (Dekker, 2014). Rather than focusing on “bad apples” at the frontline, the report recommended the development of safeguards for people’s fallibility—to “make it harder for people to do something wrong and easier for them to do it right” (Institute of Medicine, 2000, p. 2).

One such safeguard that has been highly successful across several domains has been to exploit the “collective intelligence” of groups who collaborate to solve problems well. The “wisdom of crowds” phenomenon dates back to Aristotle (see Landemore, 2012), it was later investigated by Galton (1907) and others more formally in the early 20th century (e.g., Gordon, 1924). The rise of “crowds” has since been promoted in a series of popular books (e.g., Surowiecki, 2004; Rheingold, 2007; McAfee & Brynjolfsson, 2017) and even in a short-lived crime television drama (Humphrey, 2017), and for good reason, since combining judgements from many individuals can be surprisingly accurate in prediction markets, forecasting sporting outcomes, box office success, and geopolitical and climate-related events (Escoffier & McKelvey, 2015; Hueffer, Fonseca, Leiserowitz, & Taylor, 2013; Tetlock, Mellers, Rohrbaugh, & Chen, 2014; Wolfers & Zitzewitz, 2004). The benefits of aggregation have also been identified across a range of safety-critical domains including the diagnosis of skin lesions (Kurvers, Krause, Argenziano, Zalaudek, & Wolf, 2015), the interpretation of mammograms (Wolf, Krause, Carney, Bogart, & Kurvers, 2015), diagnosis in emergency medicine (Kämmer, Hautz, Herzog, Kunina-Habenicht, & Kurvers, 2017), and matching unfamiliar faces (Balsdon, Summersby, Kemp, & White, 2018).

A useful analogy for thinking about system failures, such as medical mishaps or nuclear meltdowns, is the Swiss cheese model of errors by Reason (2000), which likens human systems to multiple slices of Swiss cheese layered on top of each other. Each defensive layer (e.g., alarms, physical barriers, automatic shutdowns) could prevent a breach from occurring, but has unintended flaws, or holes, which can all align and cause harm by allowing the hazard to pass through. One can think about the wisdom of crowds in a similar way: each rater is depicted as a slice of Swiss cheese; the fewer and smaller the holes, the more expertise one has, the less chance there is of making an error. As more people are layered on, if their decisions are independent and they approach the problem from different perspectives, then the holes will be misaligned, preventing the error from passing through. On the other hand, if the raters all have the same blind spots—where the “holes” align—then errors may slip through.

In this experiment, we extend this “wisdom of crowds” approach to fingerprint analysis by comparing the performance of individuals and crowds of professional fingerprint analysts. We test whether crowds of novice participants are as collectively wise as experts, and also evaluate the collective intelligence of the groups by comparing three different rules for aggregating people’s responses:

1. *Follow-the-majority*. Adopt the judgement with the most support in the group.
2. *Follow-the-most-confident*. Adopt the judgement with the highest confidence rating.
3. *Follow-the-most-senior*. Adopt the judgment of the most experienced examiner.

Majority and confidence rules have been used successfully in high-stakes domains such as breast and skin cancer detection (Kurvers et al., 2015), while the seniority rule is less common (Kämmer et al., 2017). Pooling the independent judgments of small groups of diagnosticians substantially increases performance relative to average individual performance, often better than the highest performing member. The best rule often depends on the size of the group, but in general, if the decisions being pooled are unbiased, diverse, and derived independently, then the collective output will typically outperform even the best member of the group (Surowiecki, 2004). All three of these decision rules are often used in practice across a range of applied contexts, but they can lead to very different outcomes. But what about fingerprint analysis? Is it more sensible to follow the majority, the most confident, or the most senior examiner?

## Methods

The methods and materials for this experiment are available and described at length on the Open Science Framework, including our experiment code, video instructions, trial sequences, de-identified data, and analysis scripts (http://tiny.cc/jbkxcz).

Thirty-six professional fingerprint examiners from the Australian Federal Police, Queensland Police Service, Victoria Police, and New South Wales Police (13 females and 23 males, mean age = 46 years, SD = 8, mean experience = 16.4 years, SD = 8.6) volunteered their time. Thirty-six novice participants (25 females and 11 males, mean age = 21.6 years, SD = 3.6, with no formal experience with fingerprints) consisting of undergraduate psychology students who participated for course credit and members of the broader communities at The University of Queensland and The University of Adelaide also volunteered their time. A novice control group is important for establishing expertise (Thompson, Tangen, & McCarthy, 2013), and allows us to examine whether more domain knowledge makes for a wiser crowd—which may not always be the case (Herzog & Hertwig, 2011).

The “crime scene” prints and their matches were collected and developed at The University of Queensland from undergraduate students who left their prints on various surfaces (e.g., wood, plastic, metal, and glass), so unlike genuine crime-scene prints, they had a known true origin (Cole, 2005). Simulated prints were dusted by a research assistant (who was trained by a qualified fingerprint expert), photographed, cropped, and isolated in the frame. A qualified expert reported that each simulated print contained sufficient information to make an identification if there was a clear comparison exemplar.

Each of the 36 fingerprint examiners was presented with the same set of 24 fingerprint pairs from the same finger (targets) and 24 highly similar pairs from different fingers (distractors) in a different random order. Each pair consisted of a crime-scene “latent” fingerprint and a fully rolled “arrest” fingerprint, and participants were asked to provide a rating on a 12-point scale ranging from 1 (Sure Different) to 12 (Sure Same). On target trials, when the prints were from the same person, ratings from 7 to 12 count as a “true positive”; on distractor trials, when the prints were from different people, ratings from 7 to 12 count as a “false positive.” The distractors were created by running each latent fingerprint through the National Australian Fingerprint Identification System—which consists of roughly 67 million fingerprints—to return the most similar exemplars from the database (see Tangen et al., 2011, for a similar methodology). On the first 44 of 48 trials (22 targets, 22 distractors), participants were given 20 s to examine the prints. On the final four trials (two targets, two distractors), they had an unlimited amount of time to make a decision. These four untimed trials were cycled across each of the fingerprint pairs across the 36 participants so that each fingerprint pair was examined by three different participants. After running these 36 fingerprint examiners through the experiment, we presented an identical set of 36 trial sequences with the same fingerprint pairs in the same order to 36 novice participants.

## Results

### Individual Performance

**Fig. 1:**
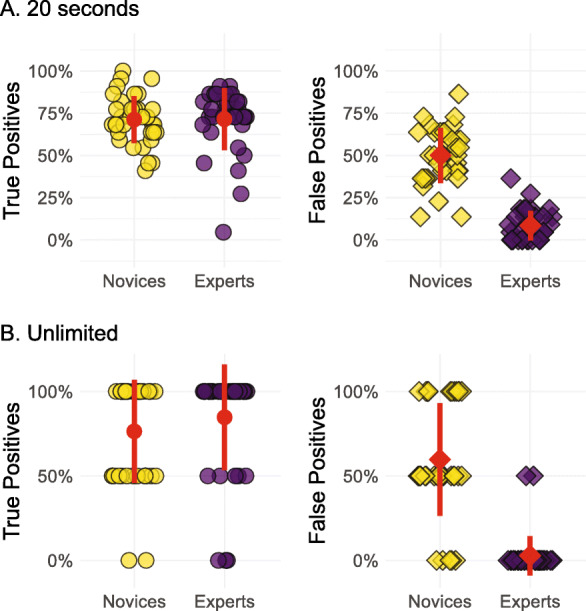
True- and false-positive rate for individual novices and experts after 20 s of analysis (**a**) or without a time limit (**b**). Each jittered data point represents the mean proportion of true or false positives for each individual participant. The *red shape* represents the mean and vertical bars ±1 standard deviation

The individual performance of the 36 novices (yellow) and 36 experts (purple) is illustrated in Fig. 1. The true-positive rate (on the left) represents each person’s performance when the prints came from the same finger. “False negatives” on these target trials are the sort of mistakes that could potentially lead to false exonerations in practice. The false-positive rate (on the right) represents each person’s performance when the prints came from different fingers. “False positives” on these distractor trials are the sort of mistakes that could potentially lead to false convictions in practice. These results closely replicate previous findings (e.g., Tangen et al., 2011; Thompson, Tangen, & McCarthy, 2014) in which experts outperformed novices on distractor trials, and performed the same or slightly better than novices on target trials. This benefit of expertise is evident in Fig. 1a during the 44 trials (22 targets, 22 distractors) in which participants were given 20 s to make a decision, and in Fig. 1b during the four trials (two targets, two distractors) in which participants had no time limit on making a decision. In the 20-s condition with 44 trials, experts made true-positive decisions 71% (SD = 45%) of the time and false-positive decisions 8.5% (SD = 28%) of the time. Novices, by comparison, made true-positive decisions 71% (SD = 45%) of the time and false-positive decisions 50% (SD = 50%) of the time. In the untimed condition with four trials, experts made true-positive decisions 85% (SD = 36%) of the time and false positives 2.8% (SD = 17%) of the time. Novices, on the other hand, made true-positive decisions 76% (SD = 43%) of the time and false positives 60% (SD = 49%) of the time.

A 2 (Group: novices vs. experts) × 2 (Rate Type: true vs. false positives) mixed ANOVA confirmed these impressions with significant main effects of Group, *F* (1, 70) = 61.34, *p* < .001, *η*_g_^2^ = .33, and Rate Type, *F* (1, 70) = 342.11, *p* < .001, *η*_g_^2^ = .68, along with a significant interaction, *F* (1, 70) = 84.25, *p* < .001, *η*_g_^2^ = .34 in the 20-s condition. The same pattern was evident in the unlimited condition: significant main effects of Group, *F* (1, 70) = 27.09, *p* < .001, *η*_g_^2^ = .16, and Rate Type, *F* (1, 35) = 110.62, *p* < .001, *η*_g_^2^ = .44, as well as a significant interaction, *F* (1, 70) = 48.47, *p* < .001, *η*_g_^2^ = .26.

### Collective Performance

The most popular, transparent, and easiest method of aggregating people’s decisions is the *majority rule* (Hastie & Kameda, 2005). It is based on the commonsense notion that “many heads are better than one,” and is commonly used when making decisions in elections and committees: choose the option that gets more than half of the votes. In the experiment described above, each of the 48 pairs of fingerprints was either judged to be from “same” or “different” fingers by 36 professional fingerprint analysts and 36 novices. For each pair of prints, we took a random sample of three analysts, and tallied the decisions made by this trio using the majority rule. We then took another random group of three analysts, tallied their decisions, and repeated this process 2000 times and for groups of 3, 5, 7, and so on for each odd group size up to 35. The result was 2000 majority decisions for each of the 48 fingerprint pairs (24 targets and 24 distractors) across the 17 different group sizes. We repeated this process for novices as well.

**Fig. 2:**
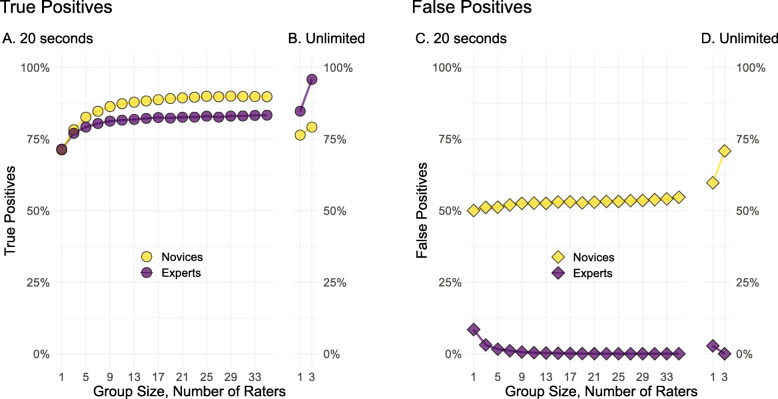
Mean true-positive rates for groups of novices and experts after 20 s of analysis (**a**) or without a time limit (**b**), and mean false positive rates for groups of novices and experts after 20 s of analysis (**c**) or without a time limit (**d**)

The results of the simulation are illustrated in Fig. 2. The individual true- and false-positive rates from Fig. 1 are represented as “Group Size, Number of Raters: 1” on the left side of each panel of Fig. 2, respectively. As we aggregate the 20-s decisions of 3, 5, 7... experts moving along the *x*-axis of Fig. 2a and c, the true-positive rate begins to increase and false-positive rate begins to decrease until they begin to level off at nine raters. For novices, however, their true-positive rate improves as more raters are included, but their false-positive rate remains at roughly 50% with a group of 35. When people are given an unlimited amount of time to decide—as illustrated in Fig. 2b and d—the benefit of expertise is even more pronounced. The true-positive rate increases from 85% for individuals to 96% for groups of three experts, but increases from 76% for individual novices to 79% for novice trios. The false-positive rate is 2.8% for individual experts, and 0% for groups of three experts. The false-positive rate for novices is 60% for individuals, and 79% for groups of three novices.

Pooling the independent judgements of a group of professional fingerprint analysts using a majority rule reduced their false-negative rate by up to 12% and their false-positive rate by up to 8%. Groups of novices, on the other hand, also received a boost in their true-positive rate of up to 19% with the majority rule, but their false-positive rate remained at roughly 50%.

**Fig. 3:**
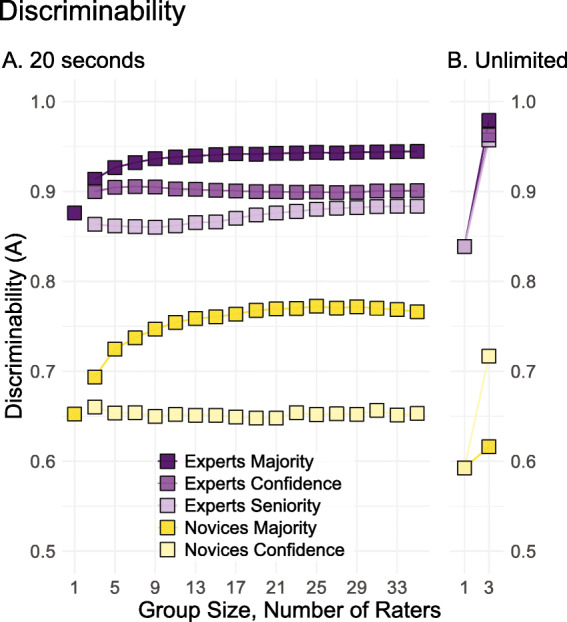
Mean discriminability scores (*A*) for experts (purple) and novices (yellow) after 20 s of analysis (**a**) or without a time limit (**b**). The *different shades of each color* represent the three aggregation rules: (1) follow-the-majority; (2) follow-the-most-confident; and (3) follow-the-most-senior

Another way to represent these results is to combine people’s true- and false-positive rates into a single measure of discriminability, which calculates how well they can distinguish between prints from the same finger and prints from different fingers. We use a non-parametric model of discriminability that averages the minimum and maximum proper receiver operating characteristic curves through a point (*A*) for each individual expert and novice participant; an *A* value of .5 is chance and 1 is perfect discriminability (Zhang & Mueller, 2005). As illustrated by the dark purple data points in Fig. 3, expert discrimination improves when taking the majority decision of small groups of examiners, leveling off at groups of nine, which is mirrored by a similar improvement by novices in dark yellow—just at a much lower level of performance.

The discriminability scores for the majority rule in Fig. 3 are presented alongside two other aggregation rules: (1) follow-the-most-confident and (2) follow-the-most-senior, which both improved the collective diagnostic performance of medical students (Kämmer et al., 2017).

We measured people’s absolute confidence on each trial by first collapsing across same and different on our 12-point scale, which ranged from 1 (Sure Different) to 12 (Sure Same), so each rating ranged from 1 (Unsure) to 6 (Sure). We then adopted the judgment with the highest confidence rating. For example, a random group of five people might provide ratings of 7, 10, 9, 8, and 1, which equates to confidence ratings of 1, 4, 3, 2, and 6. Even though four of the five provide a “Same” judgement, the extreme “Different” rating of 1 is the most extreme, so this highly confident examiner’s decision would be adopted. If people were equally confident about the two options, one was selected at random.

At the beginning of the experiment, each participant was asked to indicate how many years of formal experience they have with fingerprints. Given the follow-the-most-senior rule, we adopted the judgment of the most “senior” examiner in the crowd (i.e., the person with the greatest number of years examining prints). For example, a random group of five examiners might have 7, 10, 9, 18, and 25 years of experience. Even though the four less-experienced examiners each provide a “Same” judgement, since the most experienced examiner with 25 years of experience said, “Different,” this decision would be adopted. If examiners have the same level of experience, the response by one examiner was selected at random. Since none of the novice control participants had any experience with fingerprints, this rule was not applied to their ratings.

The output of these three aggregation rules is depicted in Fig. 3. All three rules boosted collective performance compared to individual judgements—particularly in the unlimited time condition. For novices, the majority rule produced the largest increase when given 20 s to decide, and the confidence rule produced the largest gains in the unlimited condition. But even the output of the best aggregation rule applied to novice ratings paled in comparison to experts. The majority rule produced the largest collective performance boost for experts followed by the confidence rule followed by the seniority rule—both in the 20 s and unlimited time conditions.

## Discussion

Managing errors when lives and livelihoods are at stake requires resilient systems with safeguards that can tolerate mistakes and withstand their damaging effects (Reason, 2000). The wisdom of crowds may provide one such countermeasure to mitigate their impact, which motivated us to explore the role of collective intelligence in fingerprint analysis. Our results showed that individual experts performed exceedingly well, but they still made errors. Yet when we combined their decisions using a simple majority rule, these mistakes disappeared almost entirely. Pooling the decisions from small crowds of professional fingerprint analysts makes this wise group even wiser. Pooling the decisions from small crowds of novices, however, improved their true-positive rate, but at the cost of many more false positives. We tested the effect of two other aggregation methods. The first is to adopt the decision of the most confident person in the crowd and the second is to adopt the decision of the most experienced person in the crowd. Both of these pooling methods produced a slight improvement for experts compared to individual judgements depending on the condition, but the majority rule—which is the most common, transparent, and easiest method to adopt—delivered the most considerable boost in performance.

Our results add to the growing body of evidence that combining independent judgements can greatly improve the quality of decision-making in high-stakes domains. What makes this collective intelligence approach particularly appealing in these contexts is the robustness or “fault tolerance” that is built into the aggregation process. Instead of a single examiner bearing the weight of this important decision, the burden is distributed equally across several individuals. This redundancy provides some assurance that the system will not collapse with a single mistake. Such a system would be straightforward to implement; it embodies a team-based approach to decision-making, and would bring greater peace of mind to analysts, managers, and their organizations. Of course, it is also possible that examiners could feel less responsibility for their collective decisions compared to acting alone, so they may be less conservative or careful than usual if they assume other examiners will catch their mistakes (El Zein, Bahrami, & Hertwig, 2019). Despite the promise of a collective intelligence system, courts would need to figure out how to accommodate cases where decision-making is distributed (Kemp, White, & Edmond, in press). Time and resourcing limitations could also be a consideration in adopting a distributed system, but each expert may not need to replicate the entire analysis that is currently performed by an individual examiner (Ballantyne, Edmond, & Found, 2017). Indeed, this experiment was conducted in a tightly controlled setting and should be replicated under typical conditions using actual casework materials, software tools, and timeframes. Assuming that our results generalize to everyday practice, pooling the decisions of crowds of expert analysts may provide an effective safeguard against miscarriages of justice.

## Data Availability

The data and code for each individual novice and expert participant used to produce our results and plots are available, with the exception of participants’ years of experience, which have been rank-ordered to preserve the identities of our participants.
