# Correction: Collective intelligence in fingerprint analysis

**DOI:** 10.1186/s41235-023-00514-w

**Published:** 2023-09-29

**Authors:** Jason M. Tangen, Kirsty M. Kent, Rachel A. Searston

**Affiliations:** 1 School of Psychology, The University of Queensland, St Lucia, QLD 4072 Australia; 2 School of Psychology, The University of Adelaide, Adelaide, SA 5005 Australia

**Correction: Cognitive Research: Principles and Implications (2020) 5:23** 10.1186/s41235-020-00223-8

The original article [1] contains an error in the following sentence in the Results section:

‘The false-positive rate for novices is 60% for individuals, and 79% for groups of three novices.’

The correct false-positive rate for the groups of three novices is instead 71%.
